# Antibacterial and Antibiofilm Activity of Essential Oils Against *Aeromonas* spp. Isolated from Rainbow Trout

**DOI:** 10.3390/ani14223202

**Published:** 2024-11-08

**Authors:** Patrícia Hudecová, Jana Koščová, Vanda Hajdučková, Ján Király, Peter Horňak

**Affiliations:** 1 Department of Microbiology and Immunology, University of Veterinary Medicine and Pharmacy in Košice, 041 81 Košice, Slovakia; patricia.hudecova@student.uvlf.sk (P.H.); vanda.hajduckova@uvlf.sk (V.H.); jan.kiraly@uvlf.sk (J.K.); 2 Institute of Materials and Quality Engineering, Faculty of Materials, Metallurgy and Recycling, Technical University in Košice, 040 01 Košice, Slovakia; peter.hornak@tuke.sk

**Keywords:** essential oil, *Oncorhynchus mykiss*, *Aeromonas salmonicida* subsp. *masoucida*, antimicrobial activity, biofilm

## Abstract

*Aeromonas* spp. is a major pathogen in aquaculture with a great negative economic impact. Essential oils (EOs) are compounds of the secondary metabolism of plants known for their antibacterial and antibiofilm activities. In this study, in vitro antibacterial activity of eight EOs: tea tree (extracted from *Melaleuca alternifolia*), eucalyptus (extracted from *Eucalyptus globulus* LABILL.), knee timber (extracted from *Pini mungo* L.), peppermint (extracted from *Mentha piperita* L.), oregano (extracted from *Origanum vulgare* L.), rosemary (extracted from *Rosmarinus officinalis* L.), thyme (extracted from *Thymus vulgaris* L.) and pine EO (extracted from *Pinus silvestris* L.), obtained from Calendula a.s., was evaluated. Their antibacterial activity was demonstrated against *Aeromonas* spp. isolates. Oregano and thyme EOs showed the strongest activity against all tested isolates at low concentrations, followed by tea tree and peppermint EOs. The MIC value ranged from 0.06 µL/mL to 1.0 µL/mL. The tested EOs showed a significant antibiofilm activity against biofilm-forming isolates with MBIC_50_ ranging from 0.015 µL/mL to 0.25 µL/mL. All tested isolates were obtained from rainbow trout free of clinical signs of infection. Twelve isolates of *Aeromonas salmonicida* subsp. *masoucida*, four *Aeromonas hydrophila,* and four isolates of *Aeromonas veronii* were identified. The results of the in vitro study showed a significant effect of EOs against *Aeromonas* spp., which confirmed their potential for use in aquaculture as a prevention against bacterial diseases and a way of reducing the use of antibiotics.

## Simple Summary

This study was conducted to investigate the antibacterial and antibiofilm activity of essential oils (EOs) against *Aeromonas* spp. as a major pathogen in aquaculture. EOs represent secondary metabolic compounds exhibiting strong antioxidant, antiparasitic, antiviral, or antibacterial activities, making them possible antibiotic alternatives. Of eight different EOs, oregano, extracted from *Origanum vulgare* L., and thyme EOs, extracted from *Thymus vulgaris* L., showed strong antibacterial activity against *A. salmonicida* subsp. *masoucida*, *A. veronii,* and *A. hydrophila* followed by tea tree, extracted from *Melaleuca alternifolia*, and peppermint EO, extracted from *Mentha piperita* L. Biofilm, as one of the virulence factors contributes to the ineffectiveness of antimicrobial agents and increased resistance to antibiotics. However, oregano and thyme EO have shown significant antibiofilm activity, reducing biofilm formation by *Aeromonas* isolates. These findings suggest the potential use of EOs as a preventive measure against bacterial diseases and as an antibiofilm agent in aquaculture.

## 1. Introduction

The largest sector of aquaculture is fish farming, which is also the fastest-growing food production sector. In 2023, global fisheries production increased by 16.93% to 96 million tons compared to 2018, when production was at the level of approximately 82.1 million tons [1]. Intensification of fish farming results in the development of stressful conditions for fish, such as overcrowding or unsuitable physico-chemical properties of water [2]. Intensive and semi-intensive methods in aquaculture production lead to an increased prevalence of bacterial diseases and, thus, the need for antimicrobials [3].

Bacterial pathogens of the genus *Aeromonas* are considered to be one of the major causative agents of bacterial diseases in fish farming, namely *Aeromonas salmonicida*, *Aeromonas veronii*, and *Aeromonas hydrophila*. These species cause significant economic losses in aquaculture, and due to excessive administration of antibiotics, there has been an increase in antibiotic resistance and the presence of residues in aquatic products [4]. *A. salmonicida* causes furunculosis in fish. The species includes five subspecies, of which *A. salmonicida* subsp. *salmonicida* is considered typical, while the other subsp., such as *masoucida*, *achromogenes*, *pectinolytica*, and *smithia*, represent atypical subspecies. The phenotypic difference between typical and atypical subspecies is based on different biochemical properties, growth rate, colony size, hemolytic activity, brown pigment synthesis, or origin of host isolation [5]. The infections with atypical strains manifest themselves with similar clinical signs as those with typical strains [6], such as hyperpigmentation of the skin, exophthalmia, distended abdomen, petechiae, hemorrhages at the base of the fins and forming of the typical furuncle lesions [7]. *A. veronii* is capable of causing epidemics in freshwater fish and the infection manifests itself with exophthalmia, fin rot, abdominal distention and ulceration [8], as in *Aeromonas hydrophila* infection. Infections often cause enormous damage and economic losses in rainbow trout farming [6]. The persistence of *Aeromonas* spp. in different environments and its increased antibiotic resistance can be caused by its ability to form a biofilm. As a result of its structure and composition, the difficult diffusion of antimicrobial agents through biofilms leads to the ineffectiveness of antimicrobials against bacteria [9]. Due to the global increase in antibiotic resistance, there is a need to find an effective antibiofilm agent as well as an alternative to antibiotics, and essential oils (EOs) represent the potential alternative.

EOs represent a hydrophobic mixture of secondary metabolic compounds exhibiting strong antioxidant, antiparasitic, antiviral, and antibacterial activities that help to prevent outbreaks of diseases in fish farming [10,11]. The use of EOs in aquaculture is increasing due to their biodegradability, which prevents the accumulation of their residues in tissues [12]. The mechanism of their antibacterial action consists of the disruption of electron transfer, inhibition of ATP, and disruption of the cellular wall as well as the cell membrane by inhibiting peptidoglycan synthesis or denaturation of proteins. They also affect the genetic material as they prevent DNA repair and cause inhibition of gene expression [13]. According to Da Cunha et al. [14], EOs and their active compounds have shown promising results as an alternative in the prevention of bacterial diseases in fish, and several studies have confirmed their antibiofilm potential [15,16].

In this study, we evaluated in vitro antibacterial activity of EOs against fish pathogens, *Aeromonas* spp., isolated from rainbow trout. Firstly, the disc diffusion method was used to examine eight EOs for their antibacterial activity (thyme, oregano, peppermint, tea tree, eucalyptus, knee timber, rosemary, and pine EO). Consequently, minimum inhibitory concentration (MIC) was determined for four EOs with the most effective activity, the time–kill curve was determined for thyme and oregano EOs, and their effect was observed at the morphological level by scanning electron microscopy (SEM). Finally, EOs with the strongest antibacterial activity were tested for their antibiofilm activity against isolates with confirmed biofilm formation.

## 2. Materials and Methods

### 2.1. Isolation and Identification of Aeromonas spp.

The intestinal contents of 108 rainbow trout obtained from the Slovak Republic from one breeding facility and two natural water sources were diluted with saline solution and examined by culturing using the following nutrient media: blood agar (BA) (HiMedia, Mumbai, India), Endo agar (EA) (HiMedia, India), and selective Aeromonas Agar Base (HiMedia, India). Cultivation was carried out at 25 °C for 24 h. A loopful of the pure grayish colony with oxidase and catalase activity and usually with beta-hemolytic activity on BA was subsequently Gram-stained and subjected to post-cultivation identification using the NEFERMtest 24N (Erba Lachema, Brno, Czech Republic) to determine biochemical properties of the isolates, according to manufacturer’s instructions. Identification was confirmed at the gene level using the PCR method by sequencing the 16S rRNA. Bacterial DNA was extracted from an overnight culture (18 h) using GenElute Bacterial genomic DNA Kit (Merck, Darmstadt, Germany) according to the manufacturer´s instructions, with a final elution volume of 200 µL. The purity and concentration of the extracted DNA were measured with a NanoDrop™ 8000 Spectrophotometer (Thermo Fisher Scientific, Waltham, MA, USA). Two (2) µL of bacterial DNA extract were amplified with 10 µM primers Bac27F (5′-AGAGTTTGGATCMTGGCTCAG-3′) and Univ1492R (5′-CGGTTACCTTGTTACGACTT-3′), One Taq 2X Master Mix with standard buffer (New England BioLabs, Ipswich, MA, USA) to obtain amplicons with the length of 1465 bp. Amplification conditions were as follows: initial denaturation at 95 °C for 3 min, 30 cycles of denaturation at 95 °C for 30 s, annealing at 55 °C for 30 s, and extension at 68 °C for 1 min and 40 s. The final extension was performed at 68 °C for 5 min. Consequently, PCR products were separated on 1% agarose gel in Tris-acetate-EDTA buffer (pH 7.8), sent for commercial purification and Sanger sequencing in both directions (Microsynth, Wien, Austria), and evaluated with Geneious 8.0.5 and BLASTn analysis.

### 2.2. Essential Oils

Eight EOs, i.e., tea tree EO (extracted from *Melaleuca alternifolia*), eucalyptus EO (extracted from *Eucalyptus globulus* LABILL.), knee timber EO (extracted from *Pini mungo* L.), peppermint EO (extracted from *Mentha piperita* L.), oregano EO (extracted from *Origanum vulgare* L.), rosemary EO (extracted from *Rosmarinus officinalis* L.), thymus EO (extracted from *Thymus vulgaris* L.) and pine EO (extracted from *Pinus silvestris* L.) were obtained from Calendula a.s. (Nová Ľubovňa, Slovak Republic). The main constituents of the examined EOs were determined by the manufacturer by gas chromatography/mass spectrometry (GC/MS), and their chemical composition is presented in Table 1.

### 2.3. Disc Diffusion Method

The antibacterial activity of EOs against the obtained isolates of *Aeromonas* spp. was determined by the qualitative disc diffusion method. Inocula were prepared from an overnight culture (18 h) on BA and adjusted to a turbidity corresponding to a density of 1.5 × 10^8^ CFU/mL. To perform the disc diffusion method, 100 µL of inoculum was applied to Mueller–Hinton agar (MHA) (Oxoid, Basingstoke, UK) in a Petri dish. Then, a blank paper disc, 6 mm in diameter, which was aseptically treated with 10 µL of respective EO, was placed on the surface of the agar. For the negative control, sterile tryptone soya broth (TSB) (HiMedia, India) was used instead of EO, and antibiotic ciprofloxacin (5 µg/disc) (Oxoid, UK) served as a positive control. Sensitivity to EOs was evaluated after 24 h of cultivation at a temperature of 25 °C by measuring the diameter of the inhibition zone in millimeters, and the results were evaluated using Tukey´s test. This method was performed in triplicate, and the mean ± SD was calculated for each isolate.

### 2.4. Determination of Minimum Inhibitory Concentration

The MIC of the four most effective EOs was determined by broth microdilution method using microtitration plates (Brand, Wertheim, Germany) according to the modified Ghavam et al. [17] method. As EOs are lipophilic, they were processed in order to obtain a homogeneous mixture with a water-based bacterial suspension using Tween 20 (AppliChem, Darmstadt, Germany). Bacterial inocula were prepared from an overnight culture (18 h) on blood agar and adjusted to a density of 3.0 × 10^8^ CFU/mL. The final well volume was 200 µL, of which 100 µL was bacterial inoculum and 100 µL the tested EO, which was twofold diluted with TSB to give a final concentration range of 1.0 µL/mL to 0.0078 µL/mL. TSB without EO and bacterial inoculum were used as negative controls and positive controls. The plates were incubated for 24 h at 25 °C with constant shaking, and growth inhibition was evaluated spectrophotometrically by measuring absorbance using a Biotek Synergy 2 (Merck, Germany) at 600 nm. Software Prism 8.3.0 with the Dunnett test was used for statistical evaluation. The MIC was defined as the lowest dilution with no visible growth of the microorganism (absence of turbidity). The assay was performed in triplicate, and the mean ± SD was calculated for each isolate.

### 2.5. Time–Kill Curve

Antibacterial activity of oregano and thyme EOs against *A. salmonicida* subsp. *masoucida* isolate was measured by the construction of the time–kill curve according to Barbosa et al. [18] method with appropriate modifications. Tubes with 5 mL of suspension contained the final concentration of isolate suspension adjusted to 1.5 × 10^8^ CFU/mL and EO with concentrations of 0.03 µL/mL, 0.06 µL/mL and 0.125 µL/mL. For the control group, the tube with bacterial suspension without the addition of EO was used. Aliquots (500 µL) were taken at 0, 3, 6, and 24 h and tenfold diluted with saline. Cultivation was performed on MHA at 25 °C. After incubation, the number of CFU/mL was counted, and the log CFU/mL was calculated. Three or more log_10_ reductions are required for confirmation of the bactericidal effect, and less than three log_10_ reductions represent bacteriostatic activity [19].

### 2.6. Scanning Electron Microscopy

The effect of oregano and thyme EO on the ultrastructure of the bacterial isolate was examined by electron microscopy, according to Liang et al. [20], with some modifications. A suspension of *A. salmonicida* subsp. *masoucida* isolate with a density of 1.5 × 10^8^ CFU/mL in TSB was exposed to the MIC concentration of oregano and thyme EOs. The control group was treated with the solvent only. Incubation took place at 25 °C for 18 h, after which the bacterial suspension was centrifuged at 8000 rpm for 5 min. The supernatant was discarded, and the pellet was resuspended twice in 0.1 M PBS (pH 7.3) under the same centrifugation conditions. The cells were dehydrated using ethanol with an increasing concentration (20%, 50%, 80%, and 100%) for 15 min at room temperature. Finally, ethanol was replaced with tertiary butyl alcohol for 1 h. The dried cells were gold-covered and observed by a SEM (microscope JEOL JSM-35 CF, Tokyo, Japan).

### 2.7. Testing the Ability of Biofilm Formation

All *Aeromonas* spp. isolates were tested for their ability to form a biofilm by modified O´Toole [21] method using microtitration plates. A suspension of 3.0 × 10^8^ CFU/mL was prepared in saline from an overnight culture (18 h) and added to 100 µL of TSB at a ratio of 1:1. Incubation was carried out at 25 °C for 24 h. After incubation, the medium was removed, and the content of wells was stained with 0.1% crystal violet solution, which was incubated at 20 °C for 30 min. After washing five times and subsequent drying of the wells, the cell-bound crystal violet was extracted with 30% acetic acid. The testing of the ability to form a biofilm was performed in triplicate and evaluated spectrophotometrically, measuring the absorbance at a wavelength of 550 nm using a Biotek Synergy 2 device. *Staphylococcus epidermidis* CCM 4418 (Czech collection of microorganisms, Brno, Czech Republic) was used as the reference non-biofilm forming strain.

### 2.8. Determination of Antibiofilm Activity

Inhibition of biofilm formation by oregano, thyme, tea tree, and peppermint EO was evaluated against *Aeromonas* spp. isolates with the ability to form a biofilm. The antibiofilm activity of EOs was evaluated in microtitration plates by adding 100 µL of bacterial suspension with a density of 3.0 × 10^8^ CFU/mL and 100 µL of each EO with the respective concentration range. Bacterial suspension without EO was used as a negative control. The incubation was performed at 25 °C for 24 h, and then the modified O´Toole [21] protocol for testing the biofilm activity of the isolates was followed. The assay was performed in triplicate. The ability to inhibit the biofilm formation was evaluated spectrophotometrically, measuring the absorbance at 550 nm using a Biotek Synergy 2 and Prism 8.3.0 with the Dunnett test. Minimum biofilm inhibition concentration (MBIC_50_) represents the minimum concentration of EO that can inhibit biofilm formation by 50%. The inhibition percentages for each oil were calculated using the following formula [22]:

(1) $$\mathrm{Inhibition}\;\mathrm{Percentage}=\frac{\mathrm{OD}\mathrm{negative}\mathrm{control}-\mathrm{OD}\mathrm{experimental}}{\mathrm{OD}\mathrm{negative}\mathrm{control}}\times 100$$

## 3. Results

### 3.1. Identification of Isolates from Rainbow Trout

From April 2023 to May 2024, 108 samples of intestinal contents from rainbow trout without clinical signs of infection were obtained from three different parts of the Slovak Republic. A total of 20 isolates of *Aeromonas* spp. were isolated and identified. The most abundant bacteria were *A. salmonicida* subsp. *masoucida* (twelve isolates, 60.0%), followed by four isolates of *A. hydrophila* (20.0%) and four isolates of *A. veronii* (20.0%). The identification was confirmed by PCR amplification of genomic DNA using 16S rRNA universal primers to obtain amplicons of ~1470 bp. The results of 16S rRNA gene sequencing of isolates of *A. salmonicida* subsp. *masoucida* revealed 99.72% (Isolate 4, 27, 70), 99.79% (Isolate 7, 24, 73, 86), 99.65% (Isolate 59, 63), 99.93% (Isolate 71), 99.86% (Isolate 85) and 100.0% (Isolate 84) identity with *A. salmonicida* subsp. *masoucida* JCM 7873 reference strain (GenBank accession number: NR_040829.1). *A. hydrophila* isolates revealed 99.36% (Isolate 65), 100.0% (Isolate 69), 99.58% (Isolate 72) and 99.65% (Isolate 74) identity with *A. hydrophila* ATCC 7966 (GenBank accession number: NR_074841.1) and *A. veronii* isolates revealed 99.79% (Isolate 87, 88 and 89) and 99.72% (Isolate 90) identity with the reference strain *A. veronii* JCM 7375 (GenBank accession number: NR_118947.1).

Biochemical properties were determined for each isolate and *A. hydrophila* was registered positive for oxidase, catalase, ability to ferment mannitol, trehalose, arabinose, β-galactosidase, galactose, maltose, cellobiose, and saccharose as carbon and energy sources for growth or production of γ-glutamyltransferase, *N*-acetyl-β-d-glucosaminidase and phosphatase. All isolates were negative for fermentation of ornithine, lysine, acetamide, xylose, inositol or urease, and α-galactosidase (Table 2).

*A. veronii* isolates were positive for oxidase, catalase, presence of enzymes such as β-galactosidase, *N*-acetyl-β-d-glucosaminidase or fermentation of arginine, lysine, Simmons citrate, mannitol, trehalose, and malonate. None of the isolates produced urease, phosphatase, β-glucosidase, fermented ornithine, acetamide, lactose, xylose, arabinose, and hydrolyzed aesculin (Table 2).

All *A. salmonicida* subsp. *masoucida* isolates were positive for β-glucosidase, *N*-acetyl-β-d-glucosaminidase, γ-glutamyltransferase and positively fermented mannitol, trehalose, arabinose, galactose, maltose, cellobiose, saccharose or utilized citrate. Negative results were obtained for the fermentation of lysine, acetamide, lactose, xylose, malonate, inositol, and α-galactosidase production (Table 2).

### 3.2. Antibacterial Activity of EOs

The EOs were tested for their antibacterial activity against the isolated *Aeromonas* spp. by disc diffusion method. The results of mean ± SD of the zone of inhibition included a 6 mm diameter of paper disc. The largest zones of inhibition were produced by thyme (*Thymus vulgaris* L.), oregano (*Origanum vulgare* L.), tea tree (*Melaleuca alternifolia*), and peppermint (*Mentha piperita* L.) EOs. The most effective antibacterial activity against *A. salmonicida* subsp. *masoucida* was exhibited by thyme EO, followed by oregano, tea tree, and peppermint EO. *A. hydrophila* was equally sensitive to oregano and thyme EO, followed by tea tree and peppermint EO. Strong antibacterial activity of thyme and tea tree oil was also observed against *A. veronii*, followed by peppermint and oregano EO. On the other hand, knee timber, pine, eucalyptus, and rosemary EO showed a weaker antibacterial activity.

According to Rota et al. [23], the antibacterial activity of EOs can be classified as no inhibition activity (zone of inhibition < 12 mm), moderate inhibition activity (zone of inhibition <20–12 mm), or strong inhibition activity (zone of inhibition is ≥ 20 mm). Based on this classification, oregano, thyme, tea tree, and peppermint were included among EOs with the strongest inhibition activity against all isolates of *Aeromonas* spp. Rosemary, eucalyptus, and pine showed moderate to strong inhibition activity, and knee timber showed none to moderate inhibition activity against *Aeromonas* spp. isolates. The mean ± SD of the zone of inhibition of EOs for each isolate is presented in Table 3.

Four EOs with the strongest antibacterial activity confirmed by the disc diffusion method were tested for their MIC, and EOs of thyme, tea tree, peppermint, and oregano were selected. The MIC values ranged from 0.06 µL/mL to 1.00 µL/mL. The MIC of oregano and thyme was the lowest and ranged from 0.06 µL/mL to 0.25 µL/mL, while the MIC value of tea tree and peppermint EO was higher and ranged from 0.25 µL/mL to 1.00 µL/mL. The highest sensitivity to oregano EO was exhibited by *A. salmonicida* subsp. *masoucida*, with an average MIC value of 0.10 µL/mL, followed by *A. veronii* (MIC 0.11 µL/mL) and *A. hydrophila* (MIC 0.13 µL/mL). *A. salmonicida* subsp. *masoucida* was also the most sensitive to thyme EO with an average MIC value of 0.12 µL/mL, followed by *A. hydrophila* (MIC 0.13 µL/mL) and *A. veronii* (MIC 0.25 µL/mL). The most sensitive to the tea tree EO was *A. salmonicida* subsp. *masoucida*, with an average MIC value of 0.33 µL/mL, followed by *A. hydrophila* (MIC 0.50 µL/mL) and *A. veronii* (MIC 1.00 µL/mL). *A. salmonicida* subsp. *masoucida* was also the most sensitive to peppermint EO, with an average MIC value of 0.38 µL/mL, followed by *A. hydrophila* (MIC 0.50 µL/mL) and *A. veronii* (MIC 0.88 µL/mL). MIC values for each EO are summarized in Table 4.

### 3.3. Time–Kill Curve

**Figure 1:**
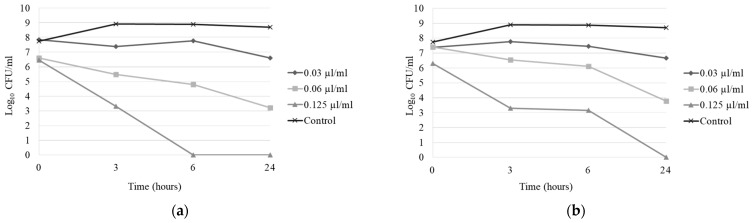
Time–kill curve against *A. salmonicida* subsp. *masoucida* isolate (**a**) with thyme EO and (**b**) oregano EO.

On the basis of the ability of EOs to inhibit pathogen growth, as determined in the previous experiment, thyme and oregano EOs were selected for further testing to determine the time–kill curve. In general, three or more log_10_ reductions in counts of bacteria indicate bactericidal activity and lower log_10_ reductions indicate the bacteriostatic ability of the antimicrobial agent [19]. After a 24-hour action, thyme EO achieved 1.6 log_10_ reduction at a concentration of 0.03 µL/mL, 2.9 log_10_ reduction at a concentration of 0.06 µL/mL (bacteriostatic activity), and 10.0 log_10_ reduction at a concentration of 0.125 µL/mL (bactericidal activity) (Figure 1a). Oregano EO exhibited strong antibacterial activity with 1.5 log_10_ reduction at a concentration of 0.03 µL/mL, 2.4 log_10_ reduction at a concentration of 0.06 µL/mL (bacteriostatic activity), and 10.0 log_10_ reduction at a concentration of 0.125 µL/mL (bactericidal activity) (Figure 1b).

### 3.4. Scanning Electron Microscopy

**Figure 2:**
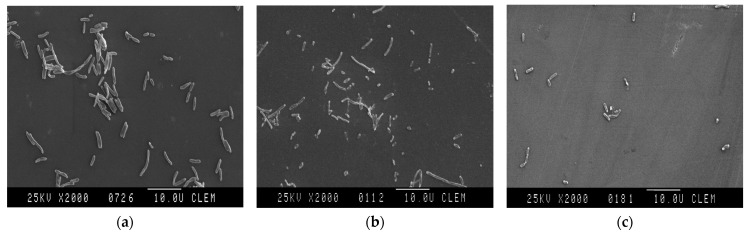
Visualization of antibacterial activity of EOs by SEM. (**a**) Visualization of the control group; bacterial suspension without EOs treatment; (**b**) antibacterial activity of thyme EO after 18 h; (**c**) and visualization of antibacterial activity of oregano EO after 18 h.

*A. salmonicida* subsp. *masoucida* isolates treated with 0.06 µL/mL concentration of oregano and thyme EO for 18 h were observed by SEM to investigate the morphological changes in the cells (Figure 2). The *A. salmonicida* subsp. *masoucida* isolate untreated with EO and used as a control showed a typical rod shape of cells that had smooth surfaces without any damage (Figure 2a). The cells treated with oregano and thyme EO showed structural damage, destruction of the cell surface, and changes in their size and shape (Figure 2b,c). Destruction of their outer membrane resulted in easy leakage of cell components up to the collapse of the cell.

### 3.5. Ability to Form a Biofilm

**Figure 3:**
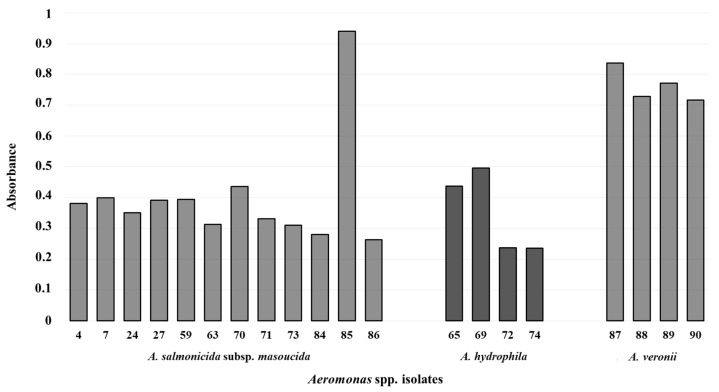
Biofilm forming by tested *Aeromonas* isolates.

The ability to form a biofilm was evaluated by the method described by Stepanovíc et al. [24], based on comparing the average OD value of individual isolates with the average OD value of the negative control (ODc = 0.441022). Subsequently, individual isolates were evaluated as biofilm non-forming (OD ≤ ODc), weak biofilm-forming (ODc < OD ≤ 2xODc), moderate biofilm-forming (2xODc < OD ≤ 4xODc), and strong biofilm-forming (4xODc < OD) isolates. Based on the results, eleven isolates of *A. salmonicida* subsp. *masoucida* (Isolates 4, 7, 24, 27, 59, 63, 70, 71, 73, 84 and 86) and three isolates of *A. hydrophila* (Isolates 65, 72 and 74) were described as non-biofilm-forming, four isolates of *A. veronii* (Isolates 87, 88, 89 and 90) and one isolate of *A. hydrophila* (Isolate 69) as weak biofilm-forming and one isolate of *A. salmonicida* subsp. *masoucida* (Isolate 85) as moderate biofilm-forming (Figure 3).

### 3.6. Antibiofilm Activity of EOs

**Figure 4:**
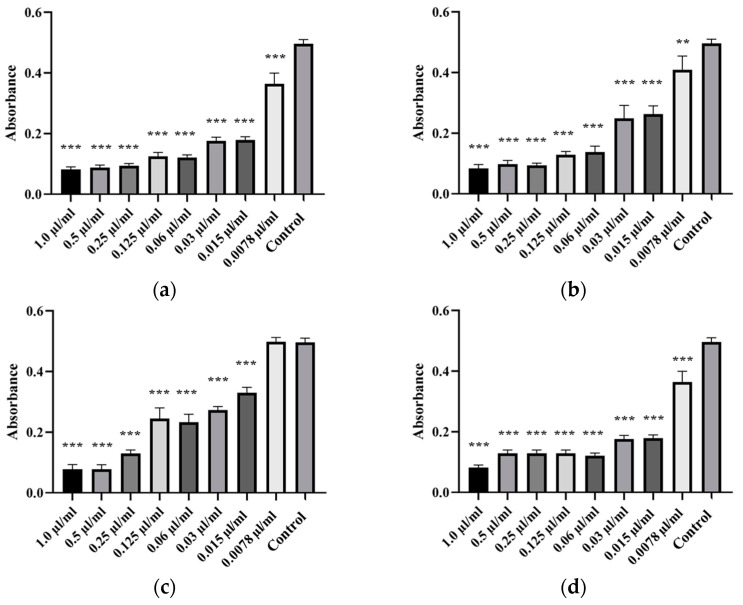
Antibiofilm activity of EOs against *A. hydrophila* isolates. Antibiofilm activity was tested against Isolate 69 (**a**) by oregano, (**b**) thyme, (**c**) tea tree, and (**d**) peppermint EO. The tested concentration of EOs ranged from 0.0078 µL/mL to 1.0 µL/mL. Statistical analysis was performed using the Dunnett test. ** *p* < 0.01, *** *p* < 0.001.

**Figure 5:**
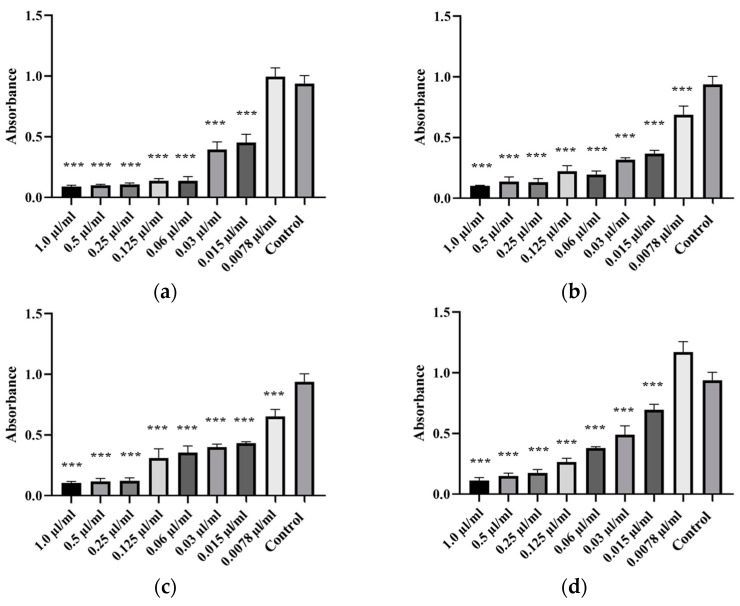
Antibiofilm activity of EOs against *A. salmonicida* subsp. *masoucida* isolate. Antibiofilm activity was tested against Isolate 85 (**a**) by oregano, (**b**) thyme, (**c**) tea tree, and (**d**) peppermint EO. The tested concentration of EOs ranged from 0.0078 µL/mL to 1.0 µL/mL. Statistical analysis was performed using the Dunnett test. *** *p* < 0.001.

**Figure 6:**
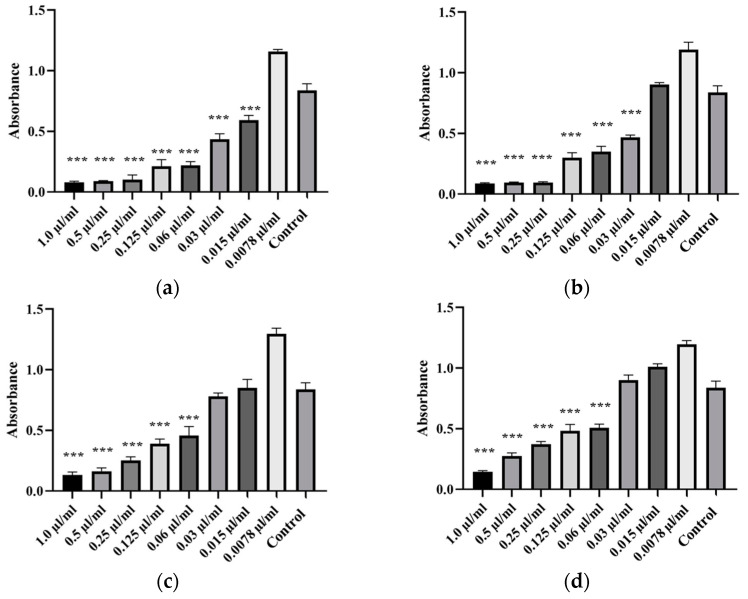

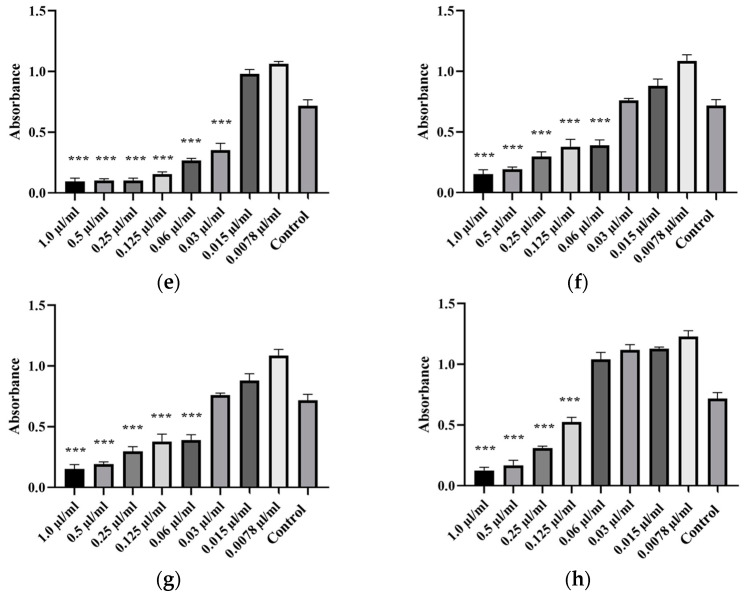
Antibiofilm activity of EOs against *A. veronii* isolates. Antibiofilm activity was tested against Isolate 87 by oregano (**a**), thyme (**b**), tea tree (**c**), and peppermint EO (**d**) and against Isolates 88, 89, and 90 by oregano (**e**), thyme (**f**), tea tree (**g**), and peppermint EO (**h**), which showed similar antibiofilm activity. The tested concentrations of EOs ranged from 0.0078 µL/mL to 1.0 µL/mL. Statistical analysis was performed using the Dunnett test. *** *p* < 0.001.

Antibiofilm properties of oregano, thyme, tea tree, and peppermint EOs were tested on weak and moderate biofilm-forming isolates, including isolates of *A. veronii* (Isolates 87, 88, 89 and 90), *A. hydrophila* (Isolate 69) and *A. salmonicida* subsp. *masoucida* (Isolate 85). Each of the four EOs showed a significant antibiofilm activity against *Aeromonas* spp. isolates. The strongest antibiofilm activity was exhibited by oregano and thyme EO, followed by tea tree and peppermint EO, while the biofilm formation increased with decreasing EO concentration. The lowest concentration of oregano EO suppressing the formation of *A. hydrophila* biofilm was 0.0078 µL/mL, for thyme EO 0.0078 µL/mL, for tea tree 0.015 µL/mL and for peppermint EO 0.0078 µL/mL (Figure 4). For *A. salmonicida* subsp. *masoucida*, the moderate biofilm-forming isolate, the lowest effective concentration of oregano EO was 0.015 µL/mL, for thyme EO 0.0078 µL/mL, for tea tree 0.0078 µL/mL and for peppermint EO 0.015 µL/mL (Figure 5). For *A. veronii* isolate, the lowest concentration of oregano EO capable of suppressing biofilm forming was 0.03–0.015 µL/mL, for thyme EO 0.03 µL/mL, for tea tree 0.06 µL/mL and for peppermint EO 0.125–0.06 µL/mL (Figure 6). The One-way ANOVA analysis revealed that the reduction in the ability to form a biofilm by isolates was significantly affected by EO concentrations (*p* < 0.05). The lowest MBIC_50_ value was determined for oregano EO, followed by thyme EO, tea tree EO, and finally peppermint EO. The MBIC_50_ values of EOs are presented in Table 5, and the ability to inhibit the biofilm formation is illustrated in Figure 4, Figure 5 and Figure 6.

## 4. Discussion

Aquaculture faces various challenges when the occurrence of fish bacterial diseases leads to high mortality rates and economic losses. Important fish pathogens include members of the genus *Aeromonas*, such as *A. hydrophila*, *A. veronii*, or *A. salmonicida* [25]. These three species have also been reported as the most prevalent *Aeromonas* species in the fish, which was also confirmed by our study. In our study, we obtained 20 *Aeromonas* spp. isolates from rainbow trout, with a 60.0% prevalence of *A. salmonicida* subsp. *masoucida*, followed by 20.0% of *A. hydrophila* and 20.0% prevalence of *A. veronii*. This is the first record of the isolation of *A. salmonicida* subsp. *masoucida* from rainbow trouts free of any clinical signs. To date, the pathogen caused great economic losses in local salmonid aquaculture in China [26]. The reported prevalence of bacteria is comparable to studies by Abd-El-Malek [27], who reported a 14.0% prevalence of *A. hydrophila* in *Oreochromis niloticus* or a 25% prevalence recorded by El Deen et al. [28]. Ebeed et al. [29] reported a 16.0% prevalence of *A. veronii* in *Oreochromis niloticus* and an 18.0% prevalence in *Mugil cephalus*. Representatives of the genus *Aeromonas* also pose a threat from the point of view of the spread of antibiotic resistance, as they have demonstrated the ability to rapidly acquire and share new resistance genes [30]. To slow down the spread of antibiotic resistance, it is necessary to look for new options, including EOs.

The use of EOs is advancing more and more, mainly in the medical and pharmaceutical industries, for their significant antimicrobial activity and potential as an alternative to antibiotics due to the rapid development of antibiotic-resistant bacteria [31]. Thanks to their beneficial properties, they are also used as food preservatives, anesthetics, antioxidants, or disinfectants [32,33,34]. On the other hand, a strong smell of some EOs may negatively affect feed intake by modulating appetite [35]. Many studies confirmed the significant antibacterial effect of EOs against Gram-positive bacteria due to their cell wall structure, which allows for easier penetration. On the other hand, the cell wall of Gram-negative bacteria contains hydrophilic lipopolysaccharides, which create a barrier for hydrophobic compounds [36]. However, a promising effect of EOs against Gram-negative bacteria was also confirmed [37,38]. Their biological activity is mainly due to the presence of volatile compounds such as esters, aldehydes, alcohols, ketones, acids, amines, camphor, myrcelon, or carvone [39]. In this study, we examined eight EOs for their antibacterial activity against Gram-negative isolates of *Aeromonas* spp. using the disc diffusion method, followed by the determination of the MIC of the four most effective EOs. Oregano, thyme, tea tree, and peppermint EO exhibited significant antibacterial activity against isolates of *A. salmonicida* subsp. *masoucida*, *A. hydrophila*, and *A. veronii*, for which MIC values ranged from 0.06 µL/mL to 1.0 µL/mL. Several studies confirmed the ability of thyme and oregano EO to inhibit the growth of multiple bacteria, such as *Staphylococcus aureus*, *E. coli* or *Campylobacter jejuni*, and *Streptococcus suis* [40,41,42]. According to Abers et al. [43], EOs of tea trees and peppermints showed promising antibacterial activity against *Streptococcus pyogenes*, *Staphylococcus aureus*, or *Klebsiella pneumoniae*. MIC values obtained in our study are comparable with other studies where MIC for oregano was 0.6 µL/mL against *Salmonella* Typhimurium, 0.3 µL/mL against *Listeria monocytogenes* and 0.055 µL/mL against *E. coli* [44,45]. Our MIC values for thyme EO are similar to those obtained by other researchers with MIC of 0.25–0.5 µL/mL [46] or 0.25 µL/mL [47] against *Staphylococcus aureus*. The antibacterial activity of tea tree EO was confirmed by de Sá Silva et al. [48], who reported that the MIC of tea tree EO against *Listeria monocytogenes* was 0.1 µL/mL. Mahboubi et al. [49] confirmed the antibacterial activity of peppermint EO with MIC 0.5 µL/mL against *Bacillus subtilis* and *Shigella dysenteriae*, and 0.25 µL/mL against *Streptococcus sobrinus*, *Streptococcus mutans* and *Bacillus cereus*. The effectiveness of the antibacterial activity of EOs may be related to their chemical composition, namely phenolic compounds such as thymol and carvacrol [50]. The antibacterial activity of oregano and thyme EO was visualized by SEM, indicating structure damage of bacterial cells as demonstrated in the study by Zhan et al. [51], in which the antibacterial activity of oregano EO caused the irregular outer surface of *Enterococcus feacalis*, or the bacteria were not uniform in size or distribution. Thyme EO showed similar antibacterial activity against *Listeria monocytogenes* that increased the permeability of cell membranes, caused cell membrane ruptures, and deformed the shape of cells, resulting in cell lysis [52]. Carvacrol and thymol, as the components of EOs, are lipophilic compounds that regulate cell walls by modifying the fatty acid profile, which affects the elasticity and permeability of the membrane. They also act as a proton exchanger, resulting in a decrease in the gradient across the cytoplasmic membrane and the depletion of the ATP pool, which leads to cell death [53].

The ability to form a biofilm is considered one of the most important factors in the pathogenesis of opportunistic bacteria [54], and aeromonads have also demonstrated the ability to adhere to biotic and abiotic surfaces with biofilm formation [55]. Of our 20 isolates, 30.0% formed a biofilm, which may increase persistence and resistance to antimicrobial agents [55]. Currently, the study of EOs deals with their potential significant antibiofilm activity; their constituents—carvacrol and thymol—show the ability to diffuse through the polysaccharide matrix of the biofilm and cause its subsequent destabilization [22]. This statement was also confirmed by our study in which oregano and thyme EOs showed significant antibiofilm activity, with a decrease in its formation in the range of EO concentration from 0.25 µL/mL to 1.0 µL/mL by 86.61%, on average. Their antibiofilm activity is also associated with the ability of thymol and carvacrol to inhibit quorum sensing (QS), which is related not only to the reduction in bacterial virulence but also to biofilm formation [53]. Tea tree EO also showed promising antibiofilm activity due to its main component, terpinen-4-ol [56], which has been shown to reduce biofilm formation by 77.18%, on average, as well as peppermint EO due to its menthol content [57] with a decrease by 72.73%, on average. Many studies confirmed the potential of EOs to be used as antibiofilm agents. Thyme EO effectively inhibited the biofilm formation of *Enterococcus faecalis* [58], *Staphylococcus aureus* [59], or *Streptococcus mutans* [60], and oregano EO showed high antibiofilm potential against *Salmonella* Typhimurium and *Listeria monocytogenes* as well [44]. According to Lui et al. [61], tea tree EO exhibited an antibiofilm activity against *Staphylococcus aureus* and *E. coli*. Peppermint EO appeared effective as an antibiofilm agent against *Staphylococcus aureus* and *Klebsiella pneumoniae* [62]. Alternative options, such as antimicrobial agents, open up new possibilities and may lead to the development of new pharmaceutical forms to improve animal and human health while slowing the spread of antibiotic resistance.

## 5. Conclusions

Nowadays, emphasis is placed on the search for substances of natural origin that could serve as alternatives to antibiotics in the context of high antibiotic resistance. This study provides information about the beneficial activities of eight EOs. Due to their properties, EOs are promising antimicrobial and antibiofilm agents. Particularly, thyme (*Thymus vulgaris* L.) and oregano EOs (*Origanum vulgare* L.) proved effective against fish pathogen *Aeromonas* spp. and also showed significant antibiofilm activity at low concentrations, followed by tea tree EO (*Melaleuca alternifolia*) and peppermint EO (*Mentha piperita* L.). Their use in aquaculture is promising and represents one of the alternative possibilities that may help to reduce outbreaks of bacterial diseases in this sector.

## Figures and Tables

**Table 1:** Chemical composition of EOs.

Essential Oil	Chemical Composition	Essential Oil	Chemical Composition
Eucalyptus EO (*Eucalyptus globulus* LABILL.)	α-pinene β-pinene sabinene α-phellandrene limonene 1.8-cineol camphor	Pine EO (*Pinus silvestris* L.)	α-pinene camphene β-pinene car-3-ene β-myrcene limonene β-phellandrene p-cymene terpinolene bornyl acetate β-caryophyllene
Knee timber EO (*Pini mungo* L.)	α-pinene camphene β-pinene car-3-ene limonene p-cymene terpinolene bornyl acetate β-caryophyllene	Rosemary EO (*Rosmarinus officinalis* L.)	α-pinene camphene β-pinene β-myrcene limonene cineol p-cymene camphor bornyl acetate α-terpineol borneol verbenone
Peppermint EO (*Mentha piperita* L.)	limonene cineol menthone menthofuran isomenthone menthyl acetate isopulegol menthol pulegone carvone cineol/limonene	Thyme EO (*Thymus vulgaris* L.)	β-myrcene γ-terpinene p-cymene linalool terpinen-4-ol thymol carvacrol
Oregano EO (*Origanum vulgare* L.)	carvacrol	Tea tree EO (*Melaleuca alternifolia*)	terpinen-4-ol

Source: Manufacturer´s datasheet.

**Table 2:** Biochemical properties of the isolates using NEFERMtest 24N.

Biochemical Test	*A. hydrophila* (*n* = 4)	*A. salmonicida* subsp. *masoucida* (*n* = 12)	*A. veronii* (*n* = 4)
	% of Positive Results		
Oxidase	100.0	100.0	100.0
Catalase	100.0	100.0	100.0
Urease	0.0	8.33	0.0
Arginine	50.0	91.67	100.0
Ornithine	0.0	8.33	0.0
Lysin	25.0	8.33	100.0
Acetamide	0.0	8.33	0.0
β-glucosidase	50.0	100.0	0.0
*N*-acetyl-β-d-glucosaminidase	100.0	100.0	100.0
Simmons citrate	75.0	58.33	100.0
Lactose	50.0	8.33	0.0
Mannitol	100.0	100.0	100.0
Trehalose	100.0	100.0	100.0
Xylose	0.0	0.0	0.0
Arabinose	100.0	50.0	0.0
α-Galactosidase	0.0	0.0	75.0
β-Galactosidase	100.0	83.33	100.0
Malonate	25.0	0.0	100.0
Galactose	100.0	100.0	75.0
Maltose	100.0	100.0	75.0
Cellobiose	100.0	66.67	25.0
Saccharose	100.0	100.0	75.0
Inositol	0.0	0.0	25.0
γ-Glutamyltransferase	100.0	100.0	75.0
Phosphatase	100.0	83.33	0.0
Aesculin	25.0	33.33	0.0

Source: Own table.

**Table 3:** Antibacterial activity of EOs determined by the disc diffusion method.

Bacterial Isolate		Oregano	Thyme	Tea Tree	Peppermint	Rosemary	Eucalyptus	Pine	Knee Timber	Ciprofloxacin
*A. salmonicida* subsp. *masoucida*	Isolate 4	38.67 ± 6.03 ^a^	46.0 ± 5.29 ^a^	26.0 ± 1.0 ^b^	24.0 ± 1.0 ^b^	23.33 ± 5.77 ^b^	17.67 ± 2.08 ^bc^	16.67 ± 2.52 ^bc^	14.0 ± 4.36 ^c^	32.67 ± 0.15
	Isolate 7	37.0 ± 4.36 ^a^	44.0 ± 1.0 ^b^	21.33 ± 1.53 ^ce^	23.0 ± 2.65 ^c^	18.67 ± 2.08 ^cef^	15.67 ± 0.12 ^de^	14.67 ± 1.53 ^df^	10.33 ± 1.15 ^d^	24.33 ± 0.12
	Isolate 24	41.67 ± 1.53 ^a^	36.0 ± 1.0 ^a^	28.67 ± 0.8 ^b^	17.67 ± 3.06 ^bd^	20.33 ± 6.81 ^bd^	17.67 ± 2.52 ^bd^	16.0 ± 1.73 ^cd^	8.33 ± 1.53 ^c^	32.0 ± 0.1
	Isolate 27	39.67 ± 0.58 ^a^	40.33 ± 4.73 ^a^	32.33 ± 5.86 ^ab^	26.67 ± 4.73 ^bc^	19.33 ± 1.15 ^ce^	16.67 ± 2.08 ^de^	17.33 ± 0.58 ^cd^	9.0 ± 1.73 ^d^	34.33 ± 0.06
	Isolate 59	39.0 ± 1.0 ^ac^	41.0 ± 1.0 ^a^	34.33 ± 2.08 ^bc^	36.0 ± 2.0 ^c^	17.33 ± 1.53 ^d^	24.33 ± 2.08 ^e^	28.67 ± 0.15 ^e^	16.67 ± 1.15 ^d^	26.33 ± 0.15
	Isolate 63	43.67 ± 2.08 ^a^	43.33 ± 1.15 ^a^	37.67 ± 1.53 ^b^	29.0 ± 2.65 ^ce^	24.0 ± 2.0 ^e^	26.67 ± 1.15 ^ce^	29.67 ± 1.53 ^c^	18.67 ± 2.08 ^d^	35.33 ± 0.12
	Isolate 70	32.67 ± 2.52 ^a^	39.33 ± 1.53 ^b^	34.0 ± 1.0 ^a^	35.0 ± 1.0 ^ab^	11.0 ± 1.73 ^e^	20.33 ± 1.53 ^c^	26.67 ± 1.53 ^d^	17.0 ± 1.0 ^c^	31.67 ± 0.12
	Isolate 71	32.0 ± 1.0 ^ac^	40.33 ± 4.73 ^b^	37.67 ± 1.53 ^ab^	30.0 ± 1.0 ^ce^	25.67 ± 2.08 ^ef^	23.67 ± 1.53 ^f^	21.67 ± 0.58 ^f^	11.67 ± 1.53 ^d^	37.33 ± 0.15
	Isolate 73	34.33 ± 1.15 ^a^	46.67 ± 0.58 ^b^	36.33 ± 2.31 ^a^	38.67 ± 2.08 ^a^	24.33 ± 1.15 ^d^	25.67 ± 2.08 ^d^	25.0 ± 1.73 ^d^	10.33 ± 0.58 ^c^	30.67 ± 0.06
	Isolate 84	42.33 ± 0.58 ^a^	43.67 ± 0.5 ^a^	22.33 ± 2.08 ^b^	28.33 ± 1.53 ^c^	23.67 ± 1.53 ^b^	26.0 ± 1.0 ^bc^	25.0 ± 3.0 ^bc^	8.33 ± 0.58 ^d^	29.67 ± 0.06
	Isolate 85	34.0 ± 1.0 ^a^	44.0 ± 1.0 ^b^	33.67 ± 1.53 ^a^	34.67 ± 1.53 ^a^	22.0 ± 1.0 ^d^	23.0 ± 2.0 ^d^	16.67 ± 1.53 ^c^	18.0 ± 1.0 ^c^	25.33 ± 0.15
	Isolate 86	33.67 ± 1.15 ^a^	33.33 ± 1.53 ^a^	39.0 ± 1.0 ^b^	31.33 ± 2.52 ^a^	16.0 ± 1.0 ^ce^	22.67 ± 2.08 ^d^	17.33 ± 1.53 ^e^	12.33 ± 1.53 ^c^	23.67 ± 0.06
*A. hydrophila*	Isolate 65	40.33 ± 1.53 ^a^	31.0 ± 1.0 ^b^	42.0 ± 2.0 ^a^	31.67 ± 2.08 ^b^	12.33 ± 1.53 ^e^	20.0 ± 1.0 ^c^	25.67 ± 1.53 ^d^	18.0 ± 2.0 ^c^	15.33 ± 0.15
	Isolate 69	24.67 ± 4.51 ^ac^	30.33 ± 1.53 ^ab^	33.67 ± 1.53 ^b^	23.67 ± 1.53 ^c^	11.33 ± 1.53 ^e^	20.67 ± 1.53 ^cd^	17.0 ± 2.65 ^de^	15.0 ± 1.0 ^de^	27.0 ± 0.17
	Isolate 72	32.67 ± 2.52 ^a^	32.0 ± 1.0 ^a^	28.67 ± 1.53 ^a^	31.0 ± 1.0 ^b^	14.33 ± 1.53 ^c^	20.0 ± 1.0 ^d^	23.33 ± 1.53 ^d^	12.67 ± 1.16 ^c^	29.33 ± 0.06
	Isolate 74	38.33 ± 0.58 ^a^	41.0 ± 1.0 ^a^	41.0 ± 1.0 ^a^	26.0 ± 2.0 ^b^	26.67 ± 0.58 ^b^	21.33 ± 0.58 ^c^	16.0 ± 1.0 ^d^	21.0 ± 1.0 ^c^	25.0 ± 0.1
*A. veronii*	Isolate 87	29.0 ± 1.0 ^a^	41.0 ± 1.0 ^b^	32.33 ± 2.52 ^ab^	35.33 ± 1.15 ^c^	26.67 ± 0.58 ^a^	20.67 ± 1.53 ^d^	16.67 ± 1.53 ^e^	21.0 ± 1.0 ^d^	29.0 ± 0.1
	Isolate 88	36.0 ± 1.0 ^ac^	40.0 ± 1.0 ^b^	39.33 ± 0.58 ^ab^	33.33 ± 2.08 ^c^	23.67 ± 1.53 ^e^	26.67 ± 1.53 ^e^	26.33 ± 1.53 ^e^	19.33 ± 1.53 ^d^	28.67 ± 0.15
	Isolate 89	28.33 ± 1.53 ^ac^	40.67 ± 1.15 ^b^	29.0 ± 1.0 ^a^	24.33 ± 2.08 ^c^	16.33 ± 0.58 ^d^	18.67 ± 1.53 ^d^	16.7 ± 1.53 ^d^	16.67 ± 1.53 ^d^	29.33 ± 0.15
	Isolate 90	30.33 ± 1.15 ^a^	43.0 ± 1.0 ^b^	35.0 ± 1.0 ^c^	33.67 ± 1.53 ^c^	18.0 ± 1.0 ^f^	23.0 ± 1.0 ^e^	21.33 ± 1.53 ^e^	13.0 ± 1.0 ^d^	24.33 ± 0.06

Legend: Values are expressed as the mean diameter of the inhibitory zone (mm) ± SD of three replicates. The diameter of the paper disc (6 mm) is included, and ciprofloxacin (5 µg/disc) was used as a positive control. Means with different letters within a row differ significantly (*p* < 0.05).

**Table 4:** Minimum inhibitory concentration (MIC) of EOs against *Aeromonas* spp.

Bacterial Isolate		Oregano	Thyme	Tea Tree	Peppermint
*A. salmonicida* subsp. *masoucida*	Isolate 4	0.06 ± 0.005	0.06 ± 0.005	0.25 ± 0.013	0.25 ± 0.007
	Isolate 7	0.06 ± 0.01	0.125 ± 0.001	0.25 ± 0.006	0.25 ± 0.011
	Isolate 24	0.06 ± 0.026	0.125 ± 0.008	0.25 ± 0.003	0.25 ± 0.0
	Isolate 27	0.06 ± 0.002	0.125 ± 0.002	0.25 ± 0.039	0.25 ± 0.049
	Isolate 59	0.125 ± 0.006	0.125 ± 0.008	0.25 ± 0.005	0.5 ± 0.001
	Isolate 63	0.125 ± 0.001	0.125 ± 0.002	0.25 ± 0.002	0.5 ± 0.005
	Isolate 70	0.125 ± 0.002	0.125 ± 0.007	0.25 ± 0.008	0.25 ± 0.004
	Isolate 71	0.125 ± 0.003	0.125 ± 0.007	0.5 ± 0.01	0.5 ± 0.001
	Isolate 73	0.06 ± 0.005	0.125 ± 0.001	0.25 ± 0.005	0.25 ± 0.006
	Isolate 84	0.125 ± 0.004	0.125 ± 0.003	0.5 ± 0.014	0.5 ± 0.003
	Isolate 85	0.125 ± 0.006	0.125 ± 0.006	0.5 ± 0.016	0.5 ± 0.018
	Isolate 86	0.125 ± 0.002	0.125 ± 0.002	0.5 ± 0.01	0.5 ± 0.003
*A. hydrophila*	Isolate 65	0.125 ± 0.002	0.125 ± 0.001	0.5 ± 0.001	0.5 ± 0.002
	Isolate 69	0.125 ± 0.003	0.125 ± 0.006	0.5 ± 0.001	0.5 ± 0.002
	Isolate 72	0.125 ± 0.008	0.125 ± 0.007	0.5 ± 0.003	0.5 ± 0.001
	Isolate 74	0.125 ± 0.003	0.125 ± 0.006	0.5 ± 0.001	0.5 ± 0.003
*A. veronii*	Isolate 87	0.06 ± 0.034	0.25 ± 0.006	1.0 ± 0.001	1.0 ± 0.004
	Isolate 88	0.125 ± 0.003	0.25 ± 0.003	1.0 ± 0.004	1.0 ± 0.003
	Isolate 89	0.125 ± 0.002	0.25 ± 0.002	1.0 ± 0.06	1.0 ± 0.02
	Isolate 90	0.125 ± 0.001	0.25 ± 0.006	1.0 ± 0.007	0.5 ± 0.02

Legend: Results are presented as means ± SD of three replicates in µL/mL (*p* < 0.05).

**Table 5:** Minimum biofilm inhibition concentration (MBIC_50_) of EOs against *Aeromonas* isolates.

Isolate	Oregano (µL/mL/%)	Thyme (µL/mL/%)	Tea Tree (µL/mL/%)	Peppermint (µL/mL/%)
*A. hydrophila* (Isolate 69)	0.015/63.94	0.06/72.2	0.06/52.99	0.015/63.94
*A. salmonicida* subsp. *masoucida* (Isolate 85)	0.015/51.95	0.015/60.92	0.015/54.11	0.06/59.65
*A. veronii* (Isolate 87)	0.03/57.4	0.06/58.28	0.125/53.54	0.25/55.37
*A. veronii* (Isolate 88)	0.03/56.84	0.06/59.27	0.06/57.43	0.25/73.23
*A. veronii* (Isolate 89)	0.06/69.76	0.06/55.55	0.25/54.6	0.25/70.32
*A. veronii* (Isolate 90)	0.03/50.7	0.03/53.11	0.25/58.6	0.25/56.78

## Data Availability

The original contributions presented in the study are included in the article, further inquiries can be directed to the corresponding author.
